# Advanced glycation of the Arg-Gly-Asp (RGD) tripeptide motif modulates retinal microvascular endothelial cell dysfunction

**Published:** 2009-08-05

**Authors:** Denise M. McDonald, Gary Coleman, Ashay Bhatwadekar, Tom A. Gardiner, Alan W. Stitt

**Affiliations:** Centre for Vision and Vascular Science, Queen’s University Belfast,Northern Ireland, UK

## Abstract

**Purpose:**

Advanced glycation endproduct (AGE) formation on the basement membrane of retinal capillaries has been previously described but the impact of these adducts on capillary endothelial cell function vascular repair remains uncertain. This investigation has evaluated retinal microvascular endothelial cells (RMECs) growing on AGE-modified fibronectin (FN) and determined how this has an impact on cell-substrate interactions and downstream oxidative responses and cell survival.

**Methods:**

RMECs were grown on methylglyoxal-modified FN (AGE-FN) or native FN as a control. RMEC attachment and spreading was quantified. In a separate treatment, the AGE-FN substrate had Arg-Gly-Asp-Ser (RGDS) or scrambled peptide added before seeding. Phosphorylation of focal adhesion kinase (FAK) and α5β1 integrin localization was assessed and apoptosis evaluated. In a subset of RMECs that remained attached to the AGE-FN substrate, the production of superoxide (O_2_^-^) was assayed using dihydroethidium (DHE) fluorescence or lucigenin, in the presence or absence of NADPH. The specificity of the O_2_^-^ assays was confirmed by inhibition in the presence of polyethylene-glycol-superoxide dismutase (PEG-SOD). AGE-mediated changes to mRNAs encoding key basement membrane proteins and regulatory enzymes were investigated using real-time RT–PCR.

**Results:**

AGE-FN reduced RMEC attachment and spreading when compared to FN controls (p<0.001). RGDS peptide enhanced cell attachment on AGE-FN (p<0.001), while the scrambled peptide had no effect. FAK phosphorylation in AGE-exposed RMECs was reduced in a time-dependent fashion, while α5β1 integrin-immunoreactivity became focal at the basal membrane. AGE-exposure induced apoptosis, a response significantly prevented by RGDS peptide. AGE-exposure caused a significant increase in basal O_2_^-^ and NADPH-stimulated production by RMECs (p<0.01), while AGE-FN also increased basement membrane associated mRNA expression (p<0.05).

**Conclusions:**

AGE substrate modifications impair the function of retinal capillary endothelium and their reparative potential in response to diabetes-related insults. Arginine-specific modifications alter vital endothelial cell interactions with the substrate. This phenomenon could play an important role in dysfunction and nonperfusion of retinal capillaries during diabetes.

## Introduction

Progressive dysfunction and depletion of the retinal microvascular endothelium eventually leads to the capillary nonperfusion that characterizes the vasodegenerative stages of diabetic retinopathy. The resultant capillary nonperfusion and ischemia is linked to sight-threatening neovascularization and macular edema in many patients. Diabetic retinopathy is a complex disorder. A wide range of pathogenic pathways have been proposed that underlie microvascular dysfunction and depletion in the diabetic retina. Among these is the irreversible formation of advanced glycation endproducts (AGEs) formed from an array of precursor molecules [1]. α-Oxaloaldehydes such as glyoxal, methylglyoxal (MGO), and 3-deoxyglucosone occur at high levels in diabetic plasma, or are significantly elevated in cells exposed to high glucose [1]. They can react directly with protein to yield intracellular and extracellular AGEs [2]. For example, MGO can give rise to the adducts Nε-(carboxyethyl)lysine (CEL) and arginine-hydroimidazolone, which have been shown to have significant pathogenic effects in cells and tissues [2,3].

In anchorage-dependent cells, such as the vascular endothelium, functionality and continued survival depends on appropriate interactions with basement membrane (BM) component proteins [4]. Detachment from this substrate ultimately results in an apoptotic death response known as anoikis [5]. Under normal conditions cellular attachment to the extracellular matrix is mediated by integrins, which serve as a direct physical link between the subcellular matrix and the intracellular actin cytoskeleton, through multiprotein complexes, known as focal adhesions [4]. Focal adhesion formation initiates subcellular localization of the signaling enzyme focal adhesion kinase (FAK) to sites of cell adhesion [4]. While growth factor and neuropeptide stimulus can induce FAK tyrosine^397^ autophosphorylation and activation of the Phosphoinositide 3-kinase (PI3-kinase), a major mode of FAK regulation is via integrin-dependent adhesion to the BM component proteins: fibronectin (FN) and collagen IV [6]. This pathway promotes cell survival via downstream phosphorylation and subsequent inactivation of several pro-apoptotic effectors, including caspase-9 [7] and the pro-apoptotic Bcl-2 homolog, BAD [8].

The proteins comprising the vascular BM are among the longest lived in the body and, as such, are highly susceptible to hyperglycemia-induced AGE modification [9]. Indeed, thickening of retinal capillary BMs is a hallmark lesion of diabetic retinopathy in diabetic animal models and patients with diabetes [10]. This pathology has been widely used as a postmortem measure of disease severity [11,12]. Diabetes also leads to a net decrease in BM proteoglycan content, which alters charge selectivity and permeability across capillary walls [13]. It has been suggested that BM modifications contribute to impaired endothelial or pericyte communication, capillary contractility, or cell interactions with constituent BM proteins [14-16].

AGEs accumulate in the retinal capillary BMs of diabetic rats [11]. These adducts on BM constituent proteins may influence normal endothelial cell function [16-18] and vascular reparative processes [19]. AGE adducts on BM also lead to impaired cell-matrix interactions and growth factor depletion in endothelial cells [20], bone marrow-derived endothelial progenitor cells (EPCs) [19], and pericytes [16,21]. This has a marked impact on pro-survival signaling in microvascular endothelial cells and ultimately leads to cell death by anoikis, possibly through specific, MGO-linked arginine modifications in ARG-GLY-ASP (RGD) and glycine-phenylalanine-hydroxyproline-glycine-glutamate-arginine (GFOGER) integrin binding sites [17]. Impaired integrin-mediated BM interactions and AGE-exposure via the basal plasma membrane has serious implications for endothelial cell function in the context of diabetic retinopathy. “Endotheliopathy” in this complication, as manifest by cell dysfunction and oxidative stress, breakdown of the blood retinal barrier, and progressive loss of capillary endothelium could be, at least in part, linked to AGE modification of the vascular BM.

In the current investigation we hypothesize that progressive modification of fibronectin by advanced glycation could contribute to diabetes-related retinal capillary endotheliopathy. Using in vitro approaches, we assess endothelial cell dynamics on a diabetic-like substrate. The data offers fresh insight into the impact of AGE-modification and in particular the relative importance of RGD recognition in retinal capillary dysfunction during progressive diabetes.

## Methods

### Preparation of AGE-modified fibronectin

Unless otherwise stated, all reagents were purchased from the Sigma Chemical Co. Ltd., (Poole, Dorset, UK). AGE-modified FN (AGE-FN) was prepared as previously described [19,22]. Briefly, four-well chamber slides were coated with FN at 2 μg/cm^2^ and incubated with MGO at 10, 50, or 100 μM or with PBS (137 mM NaCl, 2.7 mM KCl, 100 mM Na_2_HPO_4_, 2 mM KH_2_PO_4_) alone for one week at 37 °C. Following incubation, slides were extensively washed with PBS to remove MGO and unbound adducts. The levels of the MGO-derived AGE adducts argpyrimidine and EL were assayed by HPLC and competitive AGE-ELISA, respectively, and we have already reported this [19].

### Retinal microvascular endothelial cells attachment and spreading

Retinal microvascular endothelial cells (RMECs) were isolated from bovine retina by established protocols [23]. Briefly, bovine eyes were transported from a local abbatoir on ice and the retinas removed and washed free of RPE in Dulbecco's minimal essential medium (MEM; Invitrogen Life Technologies, Paisley, UK). The neural retina was then homogenized in MEM and filtered through an 87 µm filter. The trapped microvessels were digested at 37 °C for approximately 20–30 min in PBS containing 200 µg/ml pronase, 200 µg/ml DNAase, and 50 µg/ml collagenase. The filtrate was microscopically examined to determine the end point for maximum retrival of endothelial cells. Vessel fragments were then trapped in a 53 µm filter and resuspended in Dulbecco’s modified Eagles medium (DMEM) containing antibiotics (0.2 mg streptomycin sulfate, 0.12 mg benzyl penicillin and 0.2 mg kanamycin), fungizone (2.5 µg/ml) and 15% fetal calf serum (FCS; Invitrogen Life Technologies). This mixture was seeded into 25 cm^2^ Falcon flasks and maintained at 37 °C in a mixture of 5% CO_2_ and air. Endothelial cell growth was supported by DMEM containing 7.5% human platelet-poor plasma-derived serum, 5 µg/ml insulin and antibiotics. All subsequent experiments were performed on confluent monolayers of cells derived from passages 1 to 4 and plated out onto multiwell dishes. RMEC attachment was visualized in vitro by phase-contrast microscopy and quantified using manual cell counts. RMECs at a cell density of 0.9×10^4^ were seeded onto 3 cm^2^ Petri dishes (Nunc Plasticware, Rochester, NY) that had been coated with FN or AGE-FN. After 3 or 6 h, the cells were washed twice with fresh PBS to dislodge nonadherent cells after which the adherent cells were fixed in 4% paraformaldehyde (PFA) for 1 h at room temperature. Fixation was followed by washing in PBS. The number of adherent cells was counted in six separate fields of view with the aid of an eyepiece graticule.

For assessment of RMEC spreading, 0.9×10^4^ cells were allowed to attach and spread on FN or AGE-FN for 6 h at 37 °C. Following incubation, cells were visualized and designated as either “spread” or “not spread.” Spread cells were defined as those that achieved normal cell morphology similar to that found in sparse culture, while not spread cells were morphologically typified by a rounded up, phase-bright appearance. The numbers of spread and not spread cells were counted in six separate fields of view and the percentage of spread cells determined.

To assess the impact of adding “exogenous” RGD peptide, we added the Arg-Gly-Asp-Ser (RGDS) peptide and a scrambled control peptide (Ser-Asp-Gly-Arg-Gly (SDGRG)) to substrates. SDGRG does not stimulate integrins and is often used as an appropriate control peptide for RGD [24]. The peptides were added to the FN or AGE-FN substrates on 3 cm^2^ Petri dishes at concentrations of 1, 3, or 6 mM. The peptide was allowed to dry onto the substrate after which the cells were added as we have described.

### Integrin signaling

Cells were exposed to FN or AGE-FN as outlined. Following the treatment period, attached cells were then lysed on the culture dishes using lysis buffer that consisted of double distilled water containing 10 mM Tris Ph 7.2, 158 mM NaCl, 1 mM Na_3_VO_4_, 1 mM EDTA, 0.1% SDS, 1% sodium deoxycholate, 1% Triton X-100, as well as protease inhibitors (1X Complete^TM^ mini protease inhibitor cocktail; Boehringer Mannheim, Mannheim, Germany). Protein was extracted on ice and quantification of total protein (µg/µl) determined using a bicinchoninic acid (BCA) protein assay (Pierce, Rockford, IL).

An aliquot of each extracted protein sample was diluted in Laemmli buffer (containing 4% SDS; 20% glycerol; 10% 2-mercaptoethanol; 0.004% bromphenol blue in 0.125 M Tris HCl). Protein from each sample and 10 µl ProSieve^®^ molecular weight marker (Cambrex Bioscience Inc., Rockland, ME) were loaded on a denaturing 4%–20% Tris-HEPES-SDS gel (Pierce) and separated by molecular weight using sodium dodecyl sulfate PAGE (SDS–PAGE). Following SDS–PAGE, proteins were transferred onto a nitrocellulose membrane (Pall Life Sciences, Portsmouth, UK). After transfer, all washes were performed with PBS containing 0.1% Tween-20. The blocking buffer and antibody diluents contained 0.1% Tween-20 and 5% Blotto™ non-fat dry milk (Santa Cruz Biotechnology, Santa Cruz, CA). After blocking, the membrane was incubated overnight in 1:500 dilution of polyclonal primary antibody (anti-phospho-FAK (tyrosine^397^; Upstate Biotechnology). After washing at room temperature for 4×10 min, the membrane was incubated for 1 h in 1:1,000 dilution of horseradish peroxidase (HRP) labeled secondary antibody (Santa Cruz Biotechnology). HRP was detected with SuperSignal^®^ West Pico Chemiluminescent Substrate (Pierce) using a UVP^®^ AutoChemi gel analysis system equipped with Labworks^™^ 4.0 image acquisition and analysis software (UVP. Inc., Upland, CA). To calculate relative expression, we reprobed membranes with a primary monoclonal antibody to β-actin. Densitometry software (Labworks^™^ 4.0, UVP. Inc., Upland) was used to calculate expression of each protein sample relative to the density of β-actin.

### Confocal microscopy for visualization of integrin α5β1

α5β1 integrin was detected using an immunofluorescence protocol. RMECs growing on FN or AGE-FN within four-well chamber slides were fixed in 4% PFA and subsequently permeabilized with a PBS solution containing 0.1% Triton X. To reduce nonspecific binding, we incubated cells with 5% normal goat serum (NGS) overnight at 4 °C. The RMECs were then incubated with a monoclonal anti-integrin α5β1 antibody (clone HA5; Chemicon, Chandlers Ford, Hampshire, UK). This antibody has been previously used to localize this integrin combination in several cell types [25]. The antibody was diluted in PBS containing 5% NGS overnight at 4 °C after which they were washed with PBS and blocked for a further hour before incubation with a 1:1,000 dilution of Alexa-488 fluorophore labeled secondary antibody (anti-mouse Alexa-488; Invitrogen Life Technologies). After extensive washing, the cells were then incubated with 5 µg/ml propidium iodide (PI) for 20 min at room temperature to stain cell nuclei. Cells were washed extensively in PBS, mounted in Vectashield (Vector Laboratories, Peterborough, UK) and examined with a Bio-Rad Microradiance confocal scanning laser microscope (CSLM) equipped with a 25 mV Argon and 1 mV green helium-neon laser. Negative controls, in which primary antibody was omitted, ensured that the staining achieved was specific for α5β1 integrin.

### RMEC mitochondrial permeability and caspase-3 activity

Mitochondrial changes were evaluated in pre-apoptotic RMECs growing on FN or differentially modified AGE-FN (using 10, 50, and 100 uM MGO). This change was evaluated using the cationic fluorescent probe 5,5′,6,6’-tetrachloro-1,1’,3,3′-tetraethylbenzimidazolylcarbocyanie (JC-1) that assesses voltage changes in membrane potential and thus mitochondrial membrane permeability. Healthy mitochondria have the ability to concentrate the green monomeric JC-1 species (emission peak 530 nm) to yield red fluorescent J aggregates (emission peak 590 nm). Dysfunctional mitochondria, however, are unable to concentrate the monomer, and there is a net decrease in the red:green fluorescence intensity ratio. Changes in the membrane transmembrane potential RMECs using JC-1 was quantified using flow cytometry as previously described [16]. Briefly, RMECs used for these studies were cultured for 3 h on FN-coated 75 cm^2^ flasks that had been modified using 10, 50, and 100 uM MGO. Following the treatment period, JC-1 dye at a final concentration of 5 ug/ml was added to the DMEM for 30 min at 37 °C in the dark. Adherent cells were then detached via brief exposure to trypsin and versene, pelleted by light centrifugation, and resuspended in PBS at a population density of 1×10^6^/ml. Once the cells were in suspension, the JC-1 cells were immediately sorted, to exclude cell debris or doublets, using a FACSCaliber flow cytometer (Becton Dickinson, Oxford, UK) equipped with a 488 nm argon laser. Each sample was examined and gated in a forward scatter versus a side scatter dot plot. The population of RMECs showing mitochondrial permeability was determined by dividing the mean fluorescence value obtained from red fluorescence channel by the mean fluorescence value obtained from the green fluorescence channel. This allowed calculation of the red:green fluorescence intensity ratio.

As a complementary apoptosis assay, caspase-3 activity was examined using an Enzcheck^®^ Caspase-3 Assay kit (Molecular Probes, Invotrogen Life Technologies) in accordance with the manufacturer’s instructions. RMECs were cultured for 3 h on FN- or AGE-FN-coated 75 cm^2^ flasks (with or without RGD peptide coating) after which the cells were washed in PBS. Adherent cells were then detached via brief exposure to trypsin and versene, pelleted, and subsequently re-suspended in PBS at a density of 1×10^6^/ml. Next, 50 µl of 1X cell lysis buffer was added to each sample for 30 min, and the samples were centrifuged at 2.5 xg to pellet cellular debris. Afterwards, 40 µl of the supernatant from each sample was transferred into individual wells of a 96 well plate (Nunc), and 50 µl of the 1X cell lysis buffer was used as a negative control to determine substrate fluorescence. Next, 40 µl of 2X substrate working solution was added to each sample followed by incubation at room temperature for 30 min. The fluorescence of each sample (excitation=342 nm; emission=441 nm) was measured on a spectrofluorimeter (Tecan Safire, Tecan, Männedorf, Switzerland).

### Superoxide assay

Released superoxide (O_2_^-^) was measured from RMECs growing on native FN or AGE-FN with an enhanced chemiluminescence detection using lucigenin as a substrate [26]. RMECs growing on FN or AGE-FN were trypsinized. Next, 0.4×10^4^ cells from each group were added to polycarbonate luminometer tubes that contained Tyrodes-HEPES buffer. The buffer comprised the following: 140 mmol/l NaCl, 6.0 mmol/l HEPES, 2.0 mmol/l Na_2_HPO_4_, 2.0 mmol/l MgSO_4_, and 5.6 mmol/l dextrose (pH 7.40). Ten µl lucigenin was added and each sample dark adapted at room temperature for 10 min after which they were transferred to the tube housing of a Sirius luminometer (Berthold Detection Systems, Bleichstr, Germany). Luminescence was recorded in both control and AGE-FN-exposed cells in the both presence and absence of β-nicotinamide adenine dinucleotide 3′-phosphate (NADPH) and polyethylene-glycol-superoxide dismutase (PEG-SOD). Peak emission of luminescence was used to construct bar charts.

In a parallel assay, dihydroethidium (DHE) was used to assess O_2_^-^ in AGE-exposed RMECs. Cytosolic DHE exhibits blue fluorescence; however, once this probe is oxidized by O_2_^-^ to 2-hyroethidium (2-HE) and ethidium it fluoresces red [27]. 2-HE is a specific O_2_^-^-derived product and is completely inhibited by PEG-SOD. RMECs were plated onto FN-coated and AGE-FN-coated coverslips in six-well dishes (Nunc) and cultured for 24 h in 20% growth medium. After 24 h, the medium was gently aspirated, washed thrice with PBS and replaced with growth medium containing 2% serum for further 24 h incubation. For DHE staining, cells were incubated for 30 min in either Krebs-HEPES buffer, which contained 99 mM NaCl, 4.7 mM KCl, 1.2 mM MgSO_4_, 1.0 mM KH_2_PO_4_, 1.9 mM CaCl_2,_ 25 mM NaHCO_3,_ 11.1 mM glucose, and 20 mM HEPES pH 7.3, or 100 units/ml PEG-SOD that was reconstituted in Krebs-HEPES buffer. After this initial incubation, the relevant buffer was aspirated from the cells and quickly replaced with 100 µl Krebs-HEPES buffer (unstimulated) or stimulated with 100 µl 10 mM NADPH reconstituted in Krebs-HEPES. Immediately after addition of NADPH, cells were incubated in 20 µM DHE for 10 min and subsequently subjected to confocal scanning laser microscopy. The settings remained constant throughout the course of the assay. When microscopy was completed, fluorescence intensity for each sample was analyzed using Lucia G/F (Version 47) image analysis software (Nikon, Kingston upon Thames UK Ltd).

### Quantitative RT–PCR

RMECs propagated on FN or AGE-FN were harvested and subjected to total RNA extraction using the RNeasy mini kit (Qiagen, West Sussex, UK) as per the manufacturer’s instructions. The amount of RNA was quantified using a Nano drop, ND-1000 spectrophotometer (NanodropTechnologies, Wilmington, DE) by measuring absorbance at 260/280 nm. cDNA was prepared from 1 μg equivalent of the total RNA using the Sensiscript Reverse Transcription (RT) kit (Qiagen) as per the manufacturer’s instructions.

cDNA polymerase chain reaction (PCR) primers were designed using Informax’s sequence analysis and primer design software, Vector NTI (Invitrogen™ Life Technologies) from bovine DNA and RNA sequences obtained from The National Center for Biotechnology Information (NCBI, Bethesda, MD; Table 1).

**Table 1:** Primer pair sequences for real-time RT–PCR.

**Gene name (abbreviation, accession number)**	**Primer Sequence (5′-3′)**
18S	F: CTTAGAGGGACAAGTCGCG
	R: GGACATCTAAGGGCATCACA
28S	F: TTGAAAATCCGGGGGAGAG
	R: ACATTGTTCCAACATGCCAG
Matrix Metalloproteinase 1 (mmp1 **-**AF134714)	F: ACTTGTACCGGGTGGCAGCG
	R: TGGGATTTTGGGAAGGTCCG
Matrix Metalloproteinase 2 (mmp2 - NM_174745)	F: ACTTCTTCCCCCGAAAGCCC
	R: GGCACGAGCGAAGGCATCAT
Matrix Metalloproteinase 14 (mmp14 **-**NM_174390)	F: TTCCATGGTGACAGCACGCC
	R: ATGGCCCAGCTCGTGCACAG
Tissue Inhibitor Metalloproteinase 1 (timp1 - NM_174471)	sense 5′-TCCACAGGTCCCAGAACCGC-3′
	antisense 5′-CATGCTGTTCCAGGGAGCCA-3′
Tissue Inhibitor Metalloproteinase 2 (timp2 - NM_174472)	sense 5′-ACTCGATGCCCCATGATCCC-3′
	antisense 5′-GCAGGAGCCGTCGCTTCTCT-3′
Collagen IV (coll IV M63139)	sense 5′-GGTTGATGGGAGAGCCTGGC-3′
	antisense 5′-ATCACCTCTGGCCCCTGGCT-3′

Quantitative RT–PCR was performed using a LightCycler rapid thermal cycler system (Roche Diagnostics Ltd, Lewes, UK). Reactions were performed in a 10 μl volume containing 1 μl cDNA template, 0.5 μM sense and anti-sense primers, deoxyribonucleotide triphosphates (dNTPs), *Taq* DNA polymerase, buffer and SYBR Green master mix (Qiagen). A typical protocol included an initial denaturation step at 95 °C for 15 min followed by amplification of the template for 36 cycles with 95 °C denaturation for 15 s, 53 °C annealing for 15 s, and 72 °C extension for 10 s. The annealing temperature was optimized for each primer set, and the extension period depended on the length of the expected product (approximately 5 s/100 base pairs, minimum 10 s). Labeled product was detected at the end of the 72 °C extension period. To confirm amplification specificity, we subjected the PCR products from each primer to a melting curve analysis and for new primer pairs, subsequent electrophoresis on a 2% agarose gel. A Lightcycler quantification report was used to obtain average crossover values. Data was expressed in terms of ΔΔ^ct^ (relative gene expression crossover value in comparison to housekeeping mRNA) values in comparison to the control group.

### Statistical analysis

Data were expressed as the mean values ±standard error of the mean (SEM). Statistical differences in the mean were assessed using one-way ANOVA followed by the Tukey-Kramer post-hoc test for multiple comparisons unless stated specifically. All statistical analyses were performed using SPSS 14.0, (SPSS, Chicago, IL) or GraphPad InStat 3.0, (GraphPad Software, San Diego, CA). Data was considered significant at 95% (p<0.05).

## Results

### RMEC attachment and spreading

**Figure 1:**
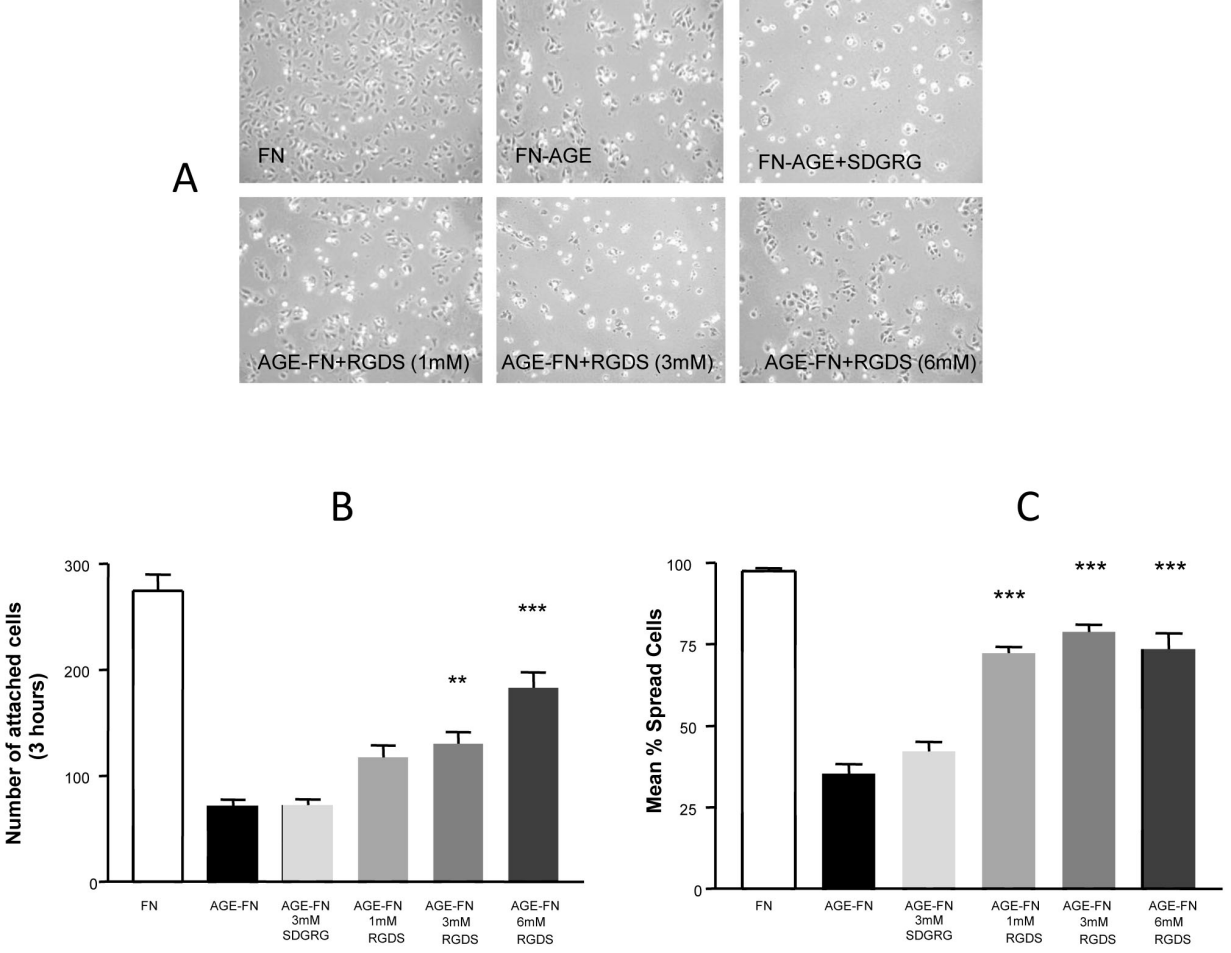
RGDS peptide modulates AGE-induced RMEC dysfunction. **A:** Phase-contrast micrographs demonstrate that AGE-FN reduces cell attachment and spreading when compared to native FN controls. RMECs cultured on AGE-FN that had been pretreated with 1–6 mM RGD peptide showed an enhanced attachment that was not evident from control (scrambled) peptide. Quantification of the RMEC responses revealed a significantly reduced attachment on AGE-FN when compared to cells cultured on native fibronectin (**B**). Supplementing AGE-FN with RGDS peptide (3 mM and 6 mM) significantly rectified cell attachment while scrambled peptide had no significant effect. A similar response was observed for RMEC spreading (**C**; n=3; **p<0.01; ***p<0.001).

RMECs seeded onto AGE-FN, which had been increasingly modified with 10, 50, and 100 µM MGO, for 3 and 6 h showed a significant step-wise reduction in attachment capacity in comparison to cells grown on native FN (p<0.001; data not shown). Using this data as a foundation, RMECs were again seeded onto AGE-FN (modified with 100 µM MGO) that had been treated with RGDS peptides or control SDGRG peptides (Figure 1) to determine if replacing the RGD motif could replenish AGE-derived loss of attachment signals. The cells demonstrated a significant reduction in attachment capacity on AGE-FN when compared to cells cultured on native FN, while supplementation with 3 mM and 6 mM RGDS peptide significantly enhanced cell attachment (p<0.01–0.001; Figure 1). The scrambled peptide had no notable effect (Figure 1). A similar response was observed for RMEC spreading responses (Figure 1).

**Figure 2:**
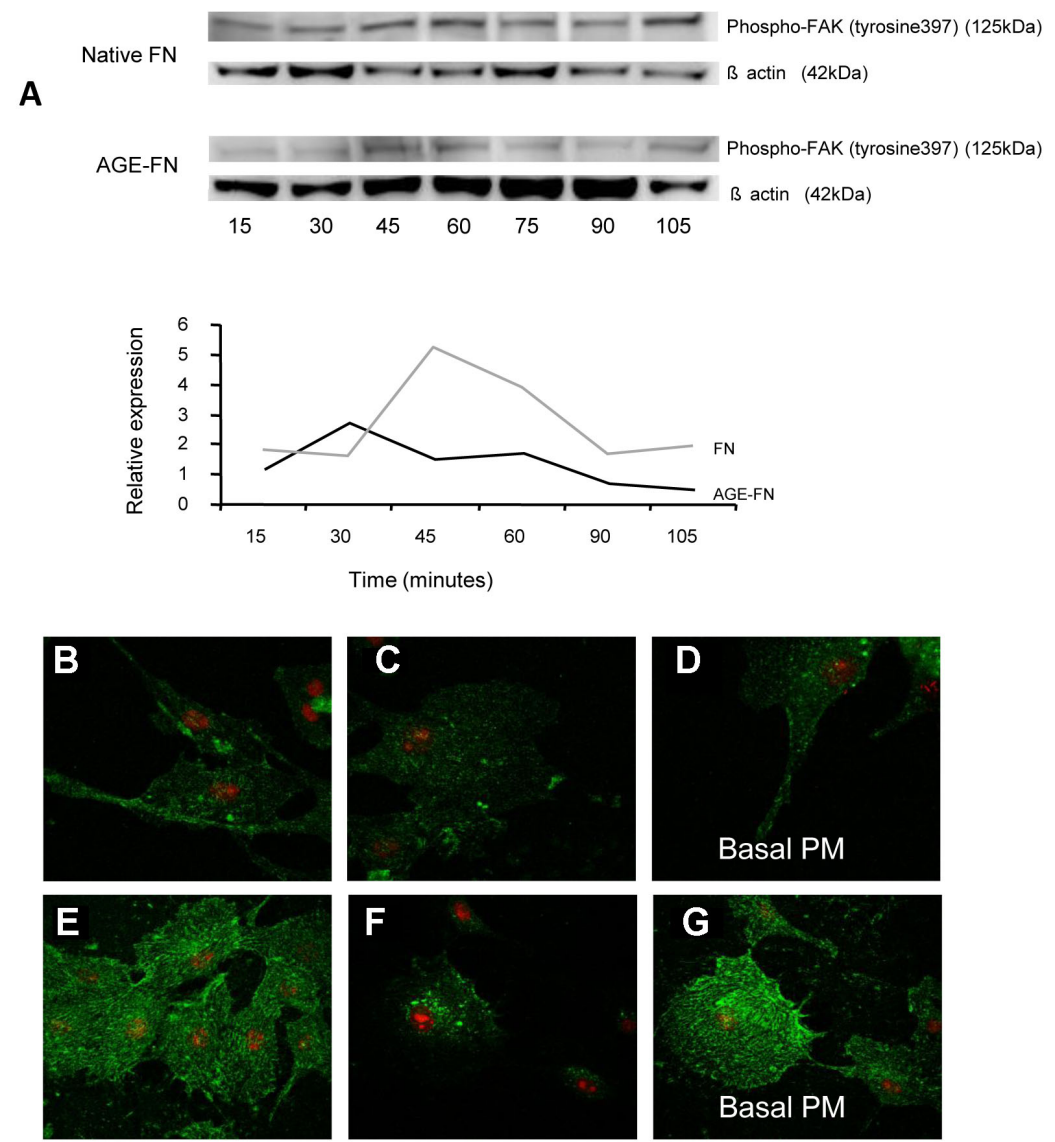
AGE-modification of FN alters integrin-mediated signaling in RMECs. **A:** western blotting analysis showed that phospho-FAK (tyrosine^397^) was reduced when RMECs were grown on AGE-FN. Quantification revealed this clear differential, which is especially evident at the 45 and 60 min time point. **B-G:** Confocal microscopy disclosed integrin α5β1 immunoreactivity in RMECs grown on FN and AGE-FN. **B-D:** RMECs cultured on native FN exhibited a relatively uniform distribution of α5β1 throughout the basal aspect of the cell immediately adjacent to the substrate. Deeper (basal) z-sections showed relatively weak fluorescence intensity at the basal plasma membrane (basal PM; **D**). **E-G:** RMECs cultured on AGE-FN exhibited a greater intensity of green fluorescence indicative of higher α5β1 expression. Some RMECs exposed to AGE-FN showed perinuclear punctuate staining (**F**). Deeper z-sections showed high fluorescence intensity and a filamentous distribution at the basal PM (**G**). All cells were counterstained with propidium iodide.

Since integrins are central to endothelial cell matrix interactions, downstream signaling responses were evaluated. RMEC signaling subsequent to attachment on FN and AGE-FN substrates were evaluated by western analysis. The levels of phospho-FAK (tyrosine^397^) were reduced when RMECs were grown on AGE-FN when compared to FN control (Figure 2A). This was especially evident at 45 min and 60 min (Figure 2A).

FAK signaling requires clustering of integrins on the plasma membrane. Investigation of integrin α5β1 immunoreactivity showed that RMECs on FN had a relatively uniform immunofluorescence immediately adjacent to the basal surface (Figure 2B). By contrast, RMECs on AGE-FN exhibited a more intense immunofluorescence for α5β1 at the basal plasma membrane and also in perinuclear organelles (compare Figure 2B,C)

### RMEC mitochondrial function and caspase-3 activity

**Figure 3:**
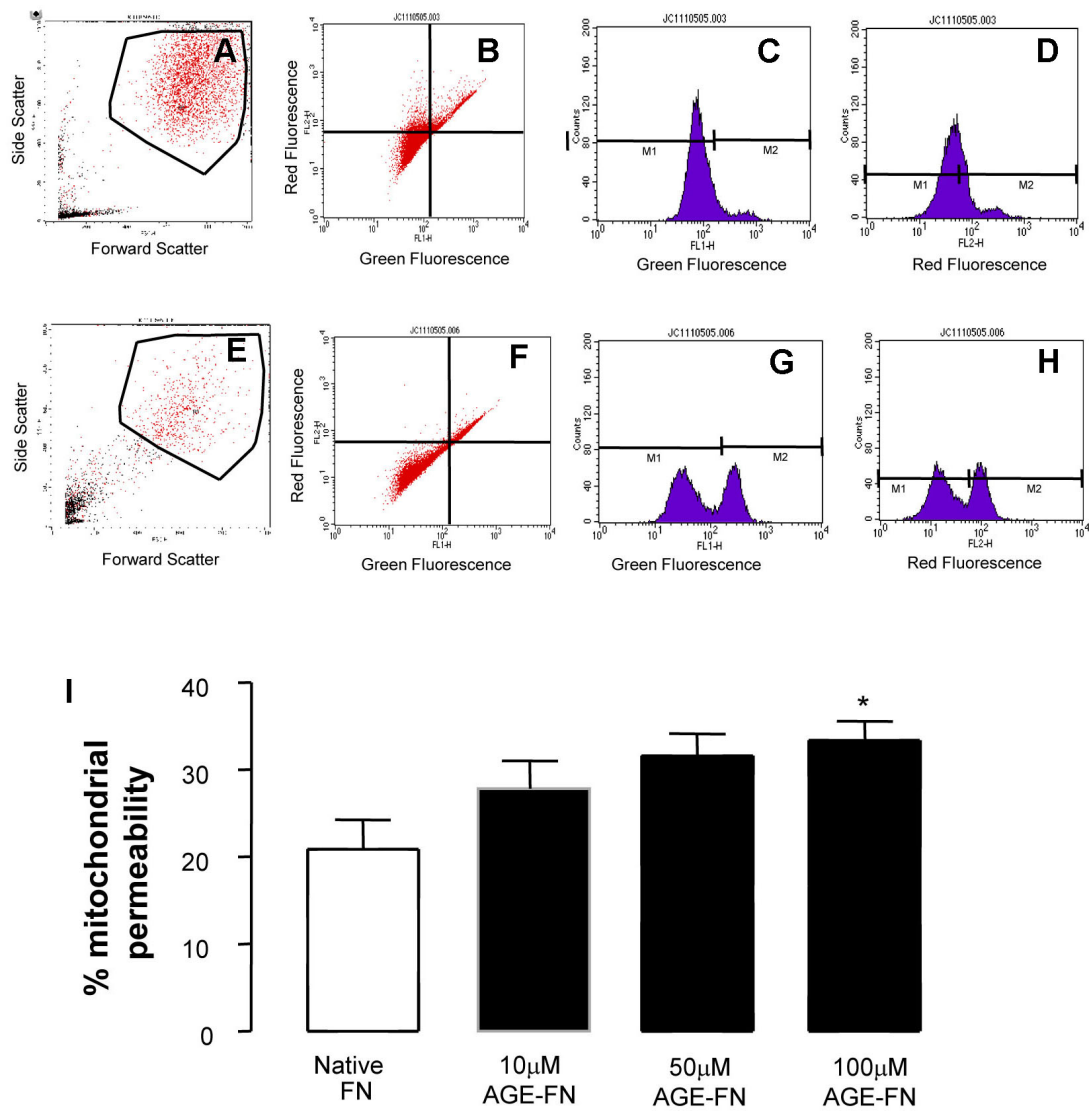
RMECs growing on AGE-FN show enhanced JC-1 mitochondrial permeability **A-D**: Cells grown on native FN (**A**) were gated to exclude cell debris in a forward scatter versus side-scatter dot plot. This dot plot is expressed in terms of both the red and green fluorescence intensity after JC-1 incubation (**B**). The histogram charts the green (**C**) and red (**D**). **E-H**: The same gating schemes were used for RMECs growing on AGE-FN. Typical traces are shown from RMECs on AGE-FN (100 µM MGO) and demonstrate that AGE-exposed cells showed shrinkage, indicating apoptosis (compare **A** with **E**). There is also a relative increase in mitochondrial permeability in AGE-exposed cells as indicated by the net decrease in the red:green fluorescence intensity ratio (**E-H**). **I:** Quantification of JC-1 fluorescence revealed that RMECs cultured on AGE-FN (modified by 10–100 µM MGO) showed a significant increase in mitochondrial permeability (red:green fluorescence intensity ratio) in comparison to cells grown on native FN. (n=3; *p<0.05; comparison between AGE-FN and native FN).

Appropriate integrin-substrate interactions are known to evoke pro-survival pathways, therefore mitochondrial permeability, as an early indicator of apoptosis, was assessed. Inner mitochondrial membrane potential was evaluated by flow cytometry using JC-1, which exhibits dual emission properties depending on mitochondrial membrane potential. As an entire population, cells grown on FN showed little mitochondrial permeability, as indicated by a constitutive red:green fluorescence intensity ratio that was significantly shifted by exposure to AGE-FN (p<0.05; Figure 3). It was apparent that AGE-exposed cells showed a degree of heterogeneity suggesting that, at this time point, some cells were more prone to mitochondrial changes than others (Figure 3).

**Figure 4:**
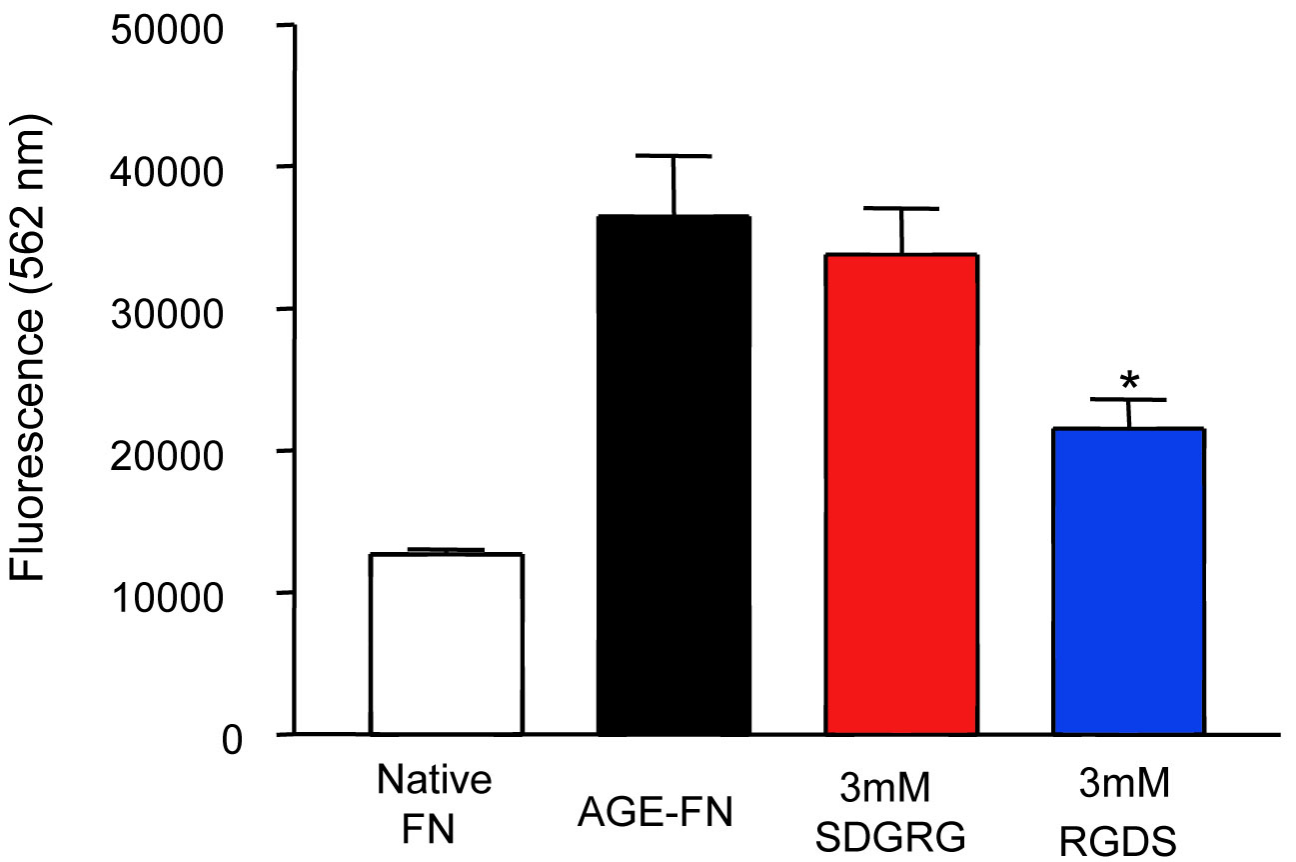
Activated caspase-3 induced by AGE-exposure is reversed by RGD peptide. RMECs growing on AGE-FN show a significant increase in caspase-3 activity. Scrambled peptide (SDGRG) plated onto the AGE-modified substrate had no influence on caspase-3 activity, while exogenous RGDS significantly reduced caspase-3 activity in RMECs cultured on AGE-FN, although this is an incomplete reduction (n=3; *p<0.05).

Caspase-3 activity in RMECs was analyzed by spectrofluorometry. This approach demonstrated that cells exposed to AGE-FN had significantly increased activity of this pro-apoptotic enzyme. Scrambled peptide plated onto the AGE-modified substrate had no influence, but RGDS peptide significantly, albeit incompletely, reduced caspase-3 activity in RMECs growing on AGE-FN (*p<0.05; Figure 4).

### Superoxide production and mRNA expression changes in RMECs

**Figure 5:**
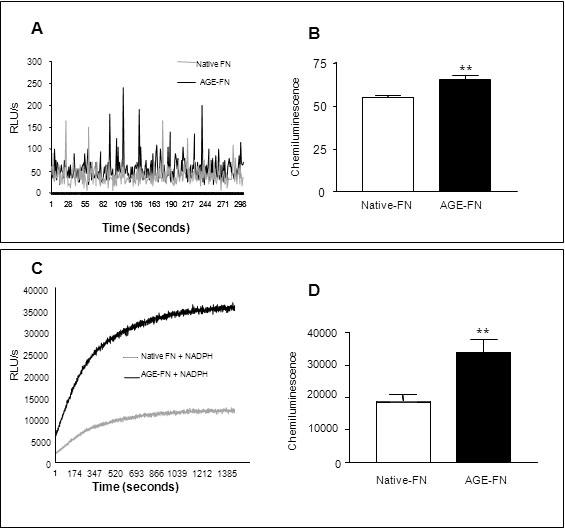
AGE-FN induce O_2_^-^ production in RMEC. The lucigenin-enhanced chemiluminescence assay was used to determine O_2_^-^ in RMECs. A typical trace is shown for RMECs growing on native FN or AGE-FN (**A**). Quantification revealed a significant increase in O_2_^-^ when cells were growing on AGE-FN (**B**). Cells stimulated with NADPH showed a significantly enhanced O_2_^-^ production (**C**) with the RMECs growing on AGE-FN, producing significantly more radicals compared to controls (**D**; n=3; **p<0.01).

O_2_^-^ generation is not only linked to many AGE-mediated cell responses [28] but is also central to endothelial cell apoptosis [29]. Following on from the attachment studies it was observed that a subset of RMEC remained attached and spread onto the AGE-modified substrate. This “surviving population” of RMECs was investigated for in situ O_2_^-^ production using the lucigenin-enhanced chemiluminescence assay. AGE-exposed cells demonstrated a significant increase in O_2_^-^ when compared to controls (p<0.01; Figure 5A,B). RMECs from both substrate groups were stimulated with NADPH. This resulted in marked O_2_^-^ production, although the AGE-exposed cells showed significantly more than RMECs growing on native FN (Figure 5C,D; **p<0.01).

**Figure 6:**
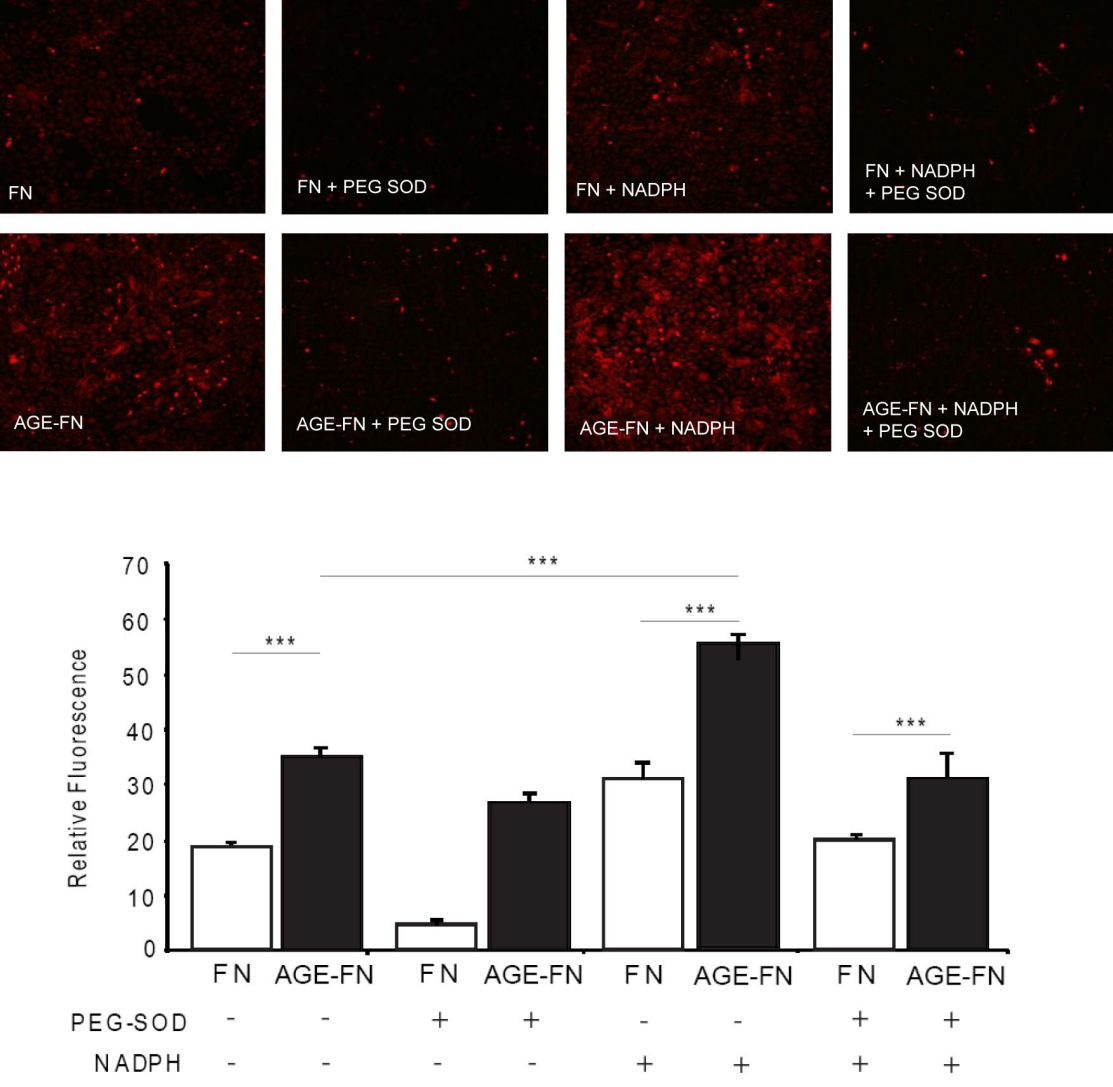
AGE-FN substrate induces O_2_^-^ in RMECs. In situ O_2_^-^ production, as measured by DHE florescence, was relatively low when RMECs were grown on FN, but this increased when the cells were exposed to NADPH. Treatment of RMECs with PEG-SOD decreased fluorescence in RMEC below basal levels, and this antioxidant treatment also significantly reduced NADPH-induced O_2_^-^. RMECs growing on AGE-FN showed higher basal levels of O_2_^-^ when compared to native FN control treatments, and this response was further increased by NADPH exposure. As with RMECs growing on FN, PEG-SOD can significantly prevent O_2_^-^ production (n=3; ***p<0.001).

As a complement to the lucigenin approach for measuring O_2_^-^ production, an assay based on DHE fluorescence was used. RMECs again showed an AGE-mediated increase in O_2_^-^ (Figure 6). RMECs growing on native FN demonstrated basal levels of O_2_^-^, but this was significantly increased when the cells were treated with NADPH (p<0.001). O_2_^-^ was attenuated with co-incubation with PEG-SOD (p<0.001; Figure 6). RMECs growing on AGE-FN produced greater levels of O_2_^-^ when compared to FN controls, and this response was significantly enhanced by NADPH exposure (p<0.01). As observed with RMECs growing on FN, PEG-SOD treatment significantly reduced NADPH-mediated O_2_^-^ production (p<0.01; Figure 6).

**Figure 7:**
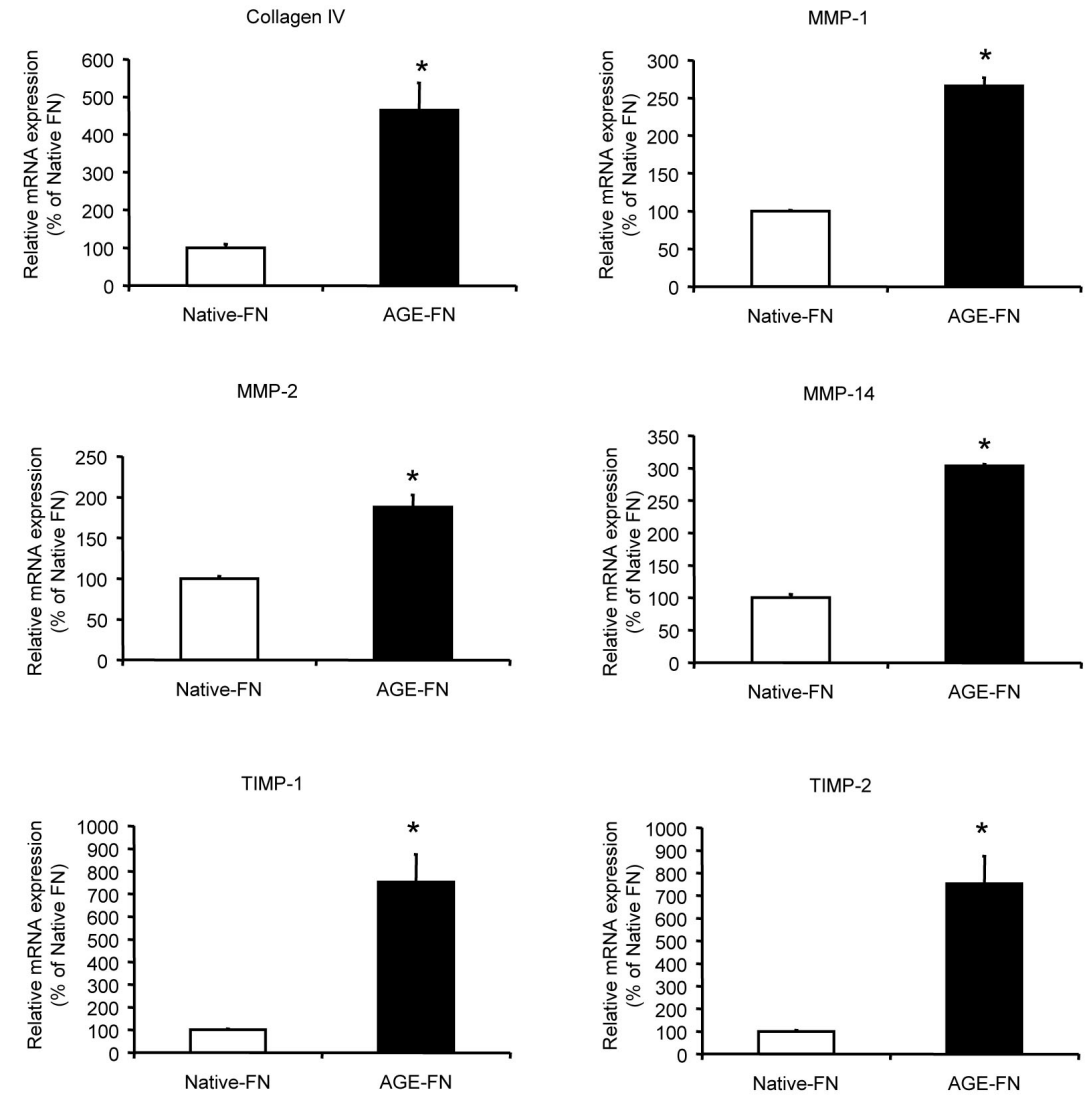
AGE-FN induces upregulation basement membrane components and associated enzymes. Endothelial cells may respond to a modified substrate by increasing synthesis of matrix proteins and enzymes that remodel the BM. Real-time RT–PCR was used to assess this response. RMECs cultured on AGE-FN displayed significant increases in BM-associated mRNA expression in comparison to RMECs cultured on Native-FN (n=3).

Previous studies have shown that AGE-modification of the BM can increase expression of the component proteins at both the protein and mRNA level, and this may contribute significantly to the BM thickening lesion [30]. In the current in vitro system it was anticipated that endothelial cells respond to an inadequate substrate by increasing synthesis of matrix proteins and enzymes that re-model the BM. Using this as a rationale, we assessed mRNA expression of the BM protein collagen IV, matrix metalloproteinases (MMP), and their regulators. Quantitative RT–PCR demonstrated that RMEC exposure to AGE-FN induced significant mRNA upregulation of collagen IV when compared to cells growing on native-FN (p<0.05; Figure 7). The proteases MMP-1, MMP-2, and MMP-14 and also the protease inhibitors tissue inhibitor of metalloproteinases-1 (TIMP-1) and TIMP-2 were also upregulated in RMECs growing on AGE-FN when compared to native FN (p<0.05; Figure 7).

## Discussion

Cell adhesion and survival is maintained via appropriate interaction of cell integrin and nonintegrin receptors with BM component proteins [4]. In this investigation, an in vitro model of AGE-modification of FN was used to study the effect of BM-immobilized adducts on retinal capillary endothelial cell function. FN was chosen because it is a major BM constituent protein and is known to accumulate AGE-modifications [31]. Furthermore, MGO is an established precursor for AGE formation with in vivo relevance and leads to modification of arginine residues in many proteins of the diabetic retina [22]. Indeed, it has been estimated that MGO-derived hydroimidazolone species could contribute up to 92% of total arginine modification by MGO [32]. Importantly, MGO is elevated in diabetic serum [33] and is involved in AGE formation in diabetic retinal capillaries [34].

The current investigation has uncovered a relationship between AGE-FN, attachment and spreading and anoikis-induced apoptosis in retinal capillary endothelium. Survival after attachment to a substrate requires not only integrin engagement, but subsequent cell spreading [35]. Failure to spread results in apoptosis by anoikis, which may be associated with retinal microvascular attenuation in human and experimental diabetes [36,37]. This study has demonstrated that AGE modification of the substrate perturbs survival-related signal transduction particularly the integrin-mediated FAK/Akt signal transduction pathway. Impaired FAK phosphorylation is consistent with a reduced bioavailability of the FN-derived RGD recognition motif. Data from the cell attachment, spreading, and apoptosis experiments show that there is a surviving subpopulation of RMEC after 6 h culture on AGE-FN. These cells appear to exhibit greater expression of the endothelial cell FN receptor integrin α5β1. Increasing both expression of α5β1 and concentrating receptor localization may increase integrin-mediated cell survival signaling through the FAK pathway and give the cells more opportunity for attachment on a diabetes-related substrate.

The RGD motif is important for cell recognition and pro-survival signaling [17,38]. Furthermore, the arginine residue of RGD is highly susceptible to MGO-derived AGE adduct formation. These modifications reduce adhesion and spreading activity of adherent cells [17,21]. Supplementation of AGE-modified BM with exogenous RGD-containing peptides was undertaken to effectively replace AGE-linked “depletion” of the RGD cell-recognition motifs on AGE-FN. This supplementation enhanced the attachment and spreading capacity of RMECs in association with a decrease in caspase-3 activation. This outcome confirms both the importance of the RGD motif in integrin-mediated cell survival and the pathogenic consequences of AGE modification of BM proteins. It should be noted that supplemented RGD peptides were not completely effective at reducing apoptosis, indicating that AGE substrates are likely to have a complex influence on cell responses and survivability. There are a range of integrin combinations, which are also likely to be important in the context of AGE-modifications of BM. Integrins such as α1 β1 and α1 β2 bind to collagen IV [39], and these interactions could also be significantly altered by exposure to AGE-modified collagen IV [17].

In the current investigation, it has been observed that a surviving subpopulation of cells remains attached and spread on the AGE-modified substrate. This is significant because although these cells remain viable it is possible that they continue to experience an AGE-mediated “insult” from the underlying substrate. It was considered that these cells could be experiencing an oxidative insult, and we evaluated the generation of O_2_^-^ by AGE-modified substrate. NADPH oxidase and NADPH oxidase-derived O_2_^-^ plays an important role in cell responses such as migration, proliferation, matrix metalloproteinase activation, and extracellular matrix synthesis [40,41]. At high concentrations, this free radical can overwhelm antioxidant defenses and lead to oxidative stress. In the diabetic state, increased glucose metabolism is associated with an increase in cellular O_2_^-^ and associated cell signaling events, although the effect of exposure to AGE-modified substrate on O_2_^-^ production has not been previously evaluated. The finding that AGE-modified substrate is linked to enhanced O_2_^-^ is a novel and important finding, suggesting that long-term modification of vascular BM during diabetes could contribute to oxidative stress in retinal capillaries.

The ability to quench fluorescence via the addition of PEG-SOD confirmed the major ROS as O_2_^-^. The NADPH stimulation outcomes indicate that the source of this radical is vascular NADPH oxidase. The actual mechanism for an O_2_^-^ AGE-linked increase is unknown but may directly involve AGE stimulation of endothelial NADPH oxidase and subsequent ROS production. Indeed, the binding of AGEs to the receptor for AGEs (RAGE) can initiate NADPH activation and subsequent production of O_2_^-^ [42], although it remains unknown if substrate-immobilized AGEs can activate RAGE in endothelial cells. This is an important issue that is beyond the scope of this manuscript but is currently under investigation.

In addition to demonstrating that RMECs cultured on AGE-FN increase O_2_^-^ production, the current study has also shown altered mRNA expression responses, particularly by enzymes that modify the BM. Several studies have shown that synthesis of BM components such as FN, laminin, and collagen IV are upregulated by high glucose or diabetes; this may be associated with the development of BM thickening [30,43,44]. Although expression of BM constituents is a pathological hallmark of diabetes and considered to be dysfunctional, it is possible that by secreting extracellular matrix, these cells are actually attempting to increase integrin-mediated cell survival via the increased production of integrin-specific cell-surface ligands. AGE-modified substrate is also associated with significant changes to mRNA expression for MMP-1, MMP-2, and MMP-14 and TIMP-1 and TIMP-2. Previous studies indicate that the ability of MMPs to modify AGE-modified matrices is compromised [45], and this could further exacerbate the consequences of diabetes-related BM modification.

It can be concluded that AGE-modification of BM component proteins significantly has an impact on the function and, ultimately, survival of the retinal microvascular endothelium. The ability of endothelium growing on such modified substrates to respond to the progressive insults incumbent in the diabetic milieu is compromised. In turn, this would impair reparative ability of the capillary endothelial monolayer. AGEs are a heterogenous group of adducts, but these findings indicate that arginine-specific modifications within extracellular proteins by MGO could play an important role in the pathogenesis of diabetic retinopathy.
